# Integrating justice in Nature-Based Solutions to avoid nature-enabled dispossession

**DOI:** 10.1007/s13280-022-01771-7

**Published:** 2022-08-24

**Authors:** Isabelle Anguelovski, Esteve Corbera

**Affiliations:** 1 Institució Catalana de Recerca I Estudis Avançats (ICREA), Passeig de Lluís Companys 23, 08010 Barcelona, Spain; 2 Institute of Environmental Science and Technology & Department of Geography, Universitat Autònoma de Barcelona, Campus UAB, 08193 Cerdanyola del Vallès, Spain

**Keywords:** Conservation, Climate adaptation policy, Climate justice, Climate mitigation policy, Land rights, Privatized nature

## Abstract

Heavily featured over the last few years in global research and policy agreements, Nature-Based Solutions (NBS) remain however exposed to much debate over the ways their current design and ability to achieve both environmental goals and social needs. As they become mainstream climate mitigation and adaptation options, their capacity to deliver expected benefits, especially when contemplating equity and justice, is at least uncertain. Through a critical review of existing debates and perspectives on NBS, this paper questions their uptake and points at the frequent embeddedness of NBS in speculative and elite-based development paths in both urban and rural areas. We present an alternative, justice-oriented approach to NBS so that projects can avoid nature-enable dispossession and instead build nature-inspired justice that prioritizes the needs, identities, and livelihoods of the most ecologically and socially vulnerable residents.

## Nature-Based Solutions as a policy panacea

While in the latest 2021 negotiations at COP-26 in Glasgow Nature-Based Solutions (NBS) heavily featured for their abundant climate co-benefits, critical scholars across the socio-environmental sciences are calling for NBS governance frameworks that can produce more just, low-carbon and adaptive societies (Toxopeus et al. 2020; Cousins 2021; Sekulova et al. 2021). Building on this emerging literature, we question the diffusion and branding of NBS as a climate savior, especially so in the context and manner in which they seem increasingly deployed—that is as a policy panacea to be enacted and scaled up through multi-stakeholder partnerships. Such partnerships are called for and enacted articulated by a variety of policy groups, climate leaders, and conservation- or urban-nature focused researchers who tend to omit or overlook the negative social impacts of NBS (Dumitru et al. 2021; European Environment Agency 2021), which can include displacement; resource, territorial, or community loss through nature commodification; and compromised long-term livelihoods (Kosoy and Corbera 2010; Kull et al. 2015; Gabriel 2016; Anguelovski et al. 2020).

In contrast, in this paper we caution against making NBS a nature-enabled dispossession for the most vulnerable residents and communities, and ask: Under which principles and conditions can NBS as a policy tool deliver justice benefits across rural and urban areas? What are promising practices that can illustrate a prioritization of justice in NBS? Upon a review of the policy context around NBS and of critical research on the topic, we propose a step-by-step approach to integrating and mainstreaming justice in NBS policy stages, one that moves away from a rather secondary, superficial, or tokenistic engagement with justice and places justice needs at the center of policy action.

NBS are actions inspired by, supported by, or copied from nature, envisioned to protect, sustainably manage, and restore ecosystems, while offering environmental, social, economic and climate resilience benefits (European Commission 2015). These actions encompass well-known land-use and climate mitigation and adaptation interventions, such as the creation of protected areas, ecological restoration and ecosystem services programs, as well as urban forestry and greening schemes, underpinned by the umbrella frameworks of ecosystem-based mitigation, adaptation or disaster risk reduction, and water-sensitive urban design or ecological engineering, among others (Dumitru et al. 2021).

As catch-all term, increasingly popular in ecology/conservation- and health-focused research as well as in policy, planning, and business circles associated with climate-centered solutions, NBS have gained incredible traction since the mid 2010s. Scholars have highlighted NBS ecosystem service provision for climate adaptation, including urban cooling and stormwater management (Gaffin et al. 2012; Baró et al. 2014; Elmqvist et al. 2016), climate mitigation gains such as carbon storage or sequestration of forests and agriculture projects (Chen 2015; De la Sota et al. 2019), and local health-related benefits (Huang et al. 2013; Wolch et al. 2014; Triguero-Mas et al. 2015). Expert groups such as the IPCC (Intergovernmental Panel on Climate Change) and IPBES (Intergovernmental Science-Policy Platform on Biodiversity and Ecosystem Services) have both advocated for the protection and restoration of nature to reduce carbon emissions, adapt to climate impacts, and protect biodiversity. In 2019, for example, the IPBES highlighted that the world faces a nature crisis with weakened capacity of nature to support our dependence on natural resources, calling for the protection and recovery of biodiversity (Intergovernmental Science-Policy Platform on Biodiversity and Ecosystem Services 2019). In 2021, the common IPBES and IPCC report pointed at the potential synergies between biodiversity conservation and climate change responses (Intergovernmental Science-Policy Platform on Biodiversity and Ecosystem Services and Intergovernmental Panel on Climate Change 2021).

Such recommendations were taken up uncritically by negotiators at the COP26 in Glasgow in November 2021, including the EU Commissioner for Environment, Oceans and Fisheries, who called nature—“our strongest ally in the fight against climate change.” (European Commission 2021). Prior to COP26, under the European Green New Deal, the European Commission (EC) had already committed to establish a larger EU-wide network of effectively managed protected areas covering 30% of land and 30% of sea, following the 2020 *Leaders’ Pledge for Nature* to reverse biodiversity loss.^1^ The EC also established a NBS-focused research-policy program aimed at advancing the upscaling of NBS, and generating evidence about their performance.^2^ Beyond policy-makers, private investors are also capitalizing upon the call for NBS, revealing the profit-making opportunities being already harvested behind putting nature at the center of climate action. For example, in a parallel side event at the COP26 Conference, the multinational law firm Clifford Chance issued a report highlighting the work of asset management firms to put NBS “into a model that’s for profit” as well as “private for-profit investment.” 3 In August 2022, Intercontinental Exchange (ICE), a global provider of data, technology, and infrastructure announced the creation of 10 Nature-Based Solutions Carbon Credit futures contracts to allow investors to purchase, sell, and hedge carbon credits from 2016 out to 2030.

In this Perspective, we suggest taking stock of both established and more recent scholarly critical evidence across the social ecological sciences, especially in human geography and planning, and avoiding the temptation of endorsing NBS as a policy panacea. This analysis also builds on the recent 2022 IPCC report which, while featuring NBS for their adaptation and mitigation benefits and contribution to other sustainable development goals, warns about the need to avoid negative impacts from NBS projects (Intergovernmental Panel on Climate Change 2022). We argue that, in their current myriad forms and applications, NBS can lead to nature-enabled dispossession and fail to deliver the conservation and climate resilience outcomes they are premised upon. By dispossession we mean here the appropriation of land, resources, and urban spaces held or enjoyed by vulnerable social groups, whose interests and relationships with nature become undermined over time, and whose own ability to remain in place become jeopardized by NBS. We stress the need to guarantee inclusive decision-making and adaptive management pathways to avoid any negative impacts resulting from NBS, such as enhanced competition for land and water with other sectors, reduction of human well-being and short term-only mitigation, pernicious investments in indigenous communities, and dynamics of gentrification through increasing land values (Intergovernmental Panel on Climate Change 2022). These impacts would expose vulnerable communities to new insecurities and impacts and exclude them from the benefits of nature conservation and climate change mitigation and adaptation. We thus challenge the extended policy and traditional scholarly view that NBS will improve “social justice, cohesion and equity” and result in more benefits than costs for the local actors being affected (Dumitru et al. 2021; European Environment Agency 2021).

Our call derives from research in sustainability science, political ecology, and environmental justice research which has demonstrated that “nature-based” policies and projects often hide environmental or sustainability “fixes” that sustain economic growth while depleting the resources and rights of historically marginalized groups (Castree 2008; Bakker 2010; Dowie 2011) as well as some of our recent research, which we integrate in our proposal of justice principles below. Recent reviews of ecosystem-based adaptation in urban and rural areas, for example, reveal projects’ insufficient attention to citizen participation and the distribution of costs and benefits across participating actors (Brink et al. 2016; Nalau et al. 2018). All in all, corporations, investors, and real estate developers have been shown to facilitate, finance, and profit from NBS to sustain natural capital valuation and profit accumulation through the commodification of nature and business-centered green urbanism (Kosoy and Corbera 2010; Kull et al. 2015; Gabriel 2016).

Over the past three decades, in rural regions, protected areas, Payments for Ecosystem Services, and the United Nations’ Clean Development Mechanism and REDD + (reducing emissions from deforestation and forest degradation) programs have been associated to net-zero emission goals that are meant to offset emissions through biodiversity conservation, ecosystem restoration, forest management, and large-scale tree planting. However, such initiatives have been critiqued for miscalculating mitigation benefits and for their inability to address concerns related to the rights, resources, and livelihoods of local communities, particularly if meaningful recognition and local participation have not guided design and implementation (Pascual et al. 2014; Oldekop et al. 2016; Pritchard and Brockington 2019; Almanza-Alcalde et al. 2021). The fact that large food conglomerates, airlines, or energy companies have invested in such schemes does not justify holding these actors unaccountable and leaving mostly industry- and resource-extraction and consumption, development pathways unaddressed.

In urban regions, the deployment of NBS as a specific strategy has gained traction more recently, particularly since the mid 2010s. Through projects such as (re)constructed wetlands, rain gardens, resilient parks, green roofs, community gardens, or waterfront clean and restoration, cities are working to bring nature back to urban residents while addressing biodiversity loss, water and air pollution, and climate threats (Kotsila et al. 2021). Yet, the increasing reliance of municipalities on privately-funded NBS, branding of new business opportunities for NBS, as well as the process of large-scale, real estate development alongside these new re-naturing areas have raised doubts about their ability to ensure social and environmental sustainability and deliver justice goals (Kabisch et al. 2016; Sekulova et al. 2021). Urban greening so far often fails to recognize and redress long-standing inequalities or to integrate different socio-cultural views and identities as related to nature (Tozer et al. 2020). Researchers have also identified green gentrification and displacement together with green rent seeking and dispossession in many cities, including Barcelona, Boston, Montreal, or Copenhagen, driven by the increasingly private sector-led as well as growth- and profit-oriented orchestration of urban NBS (I. Anguelovski et al. 2019a, b; Anguelovski et al. 2022; García-Lamarca et al. 2022).

## Toward nature-inspired justice

**Fig. 1:**
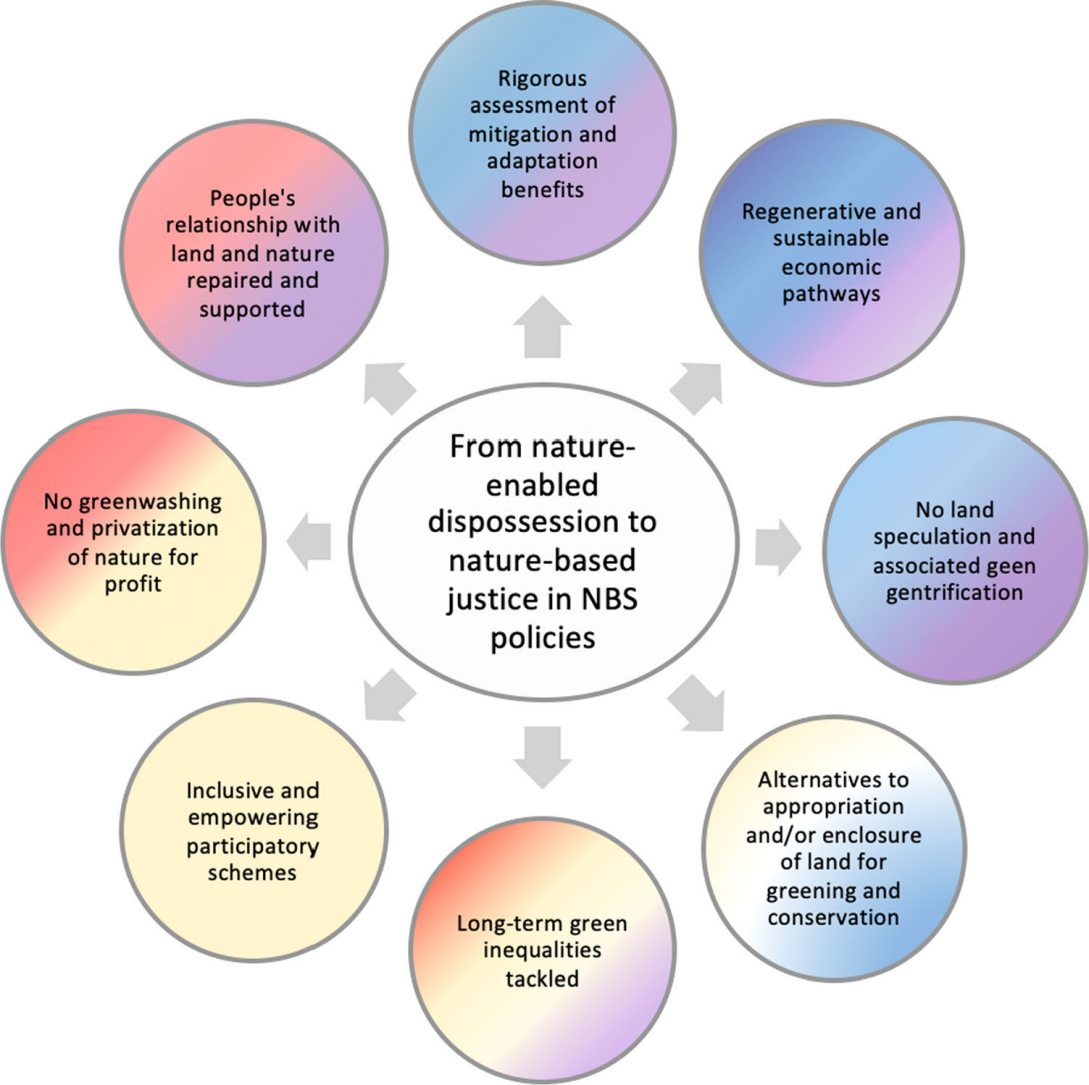
From nature-enabled dispossession to nature-inspired justice in NBS policies. Nature-based justice requires a series of principles and associated practices for tackling existing environment and climate, social, and economic challenges related to NBS, from policy option (blue), decision-making (yellow), implementation (red), to evaluation (purple), with an assumption that the principles we outline may have an overlap in the stages that tackle them, hence the use of gradients. NB: The stages of identifying the environmental problem and setting the policy agenda have been omitted because NBS are already a well-established choice in policy forums and schemes to address global environmental challenges

To avoid the types of socio-environmental dispossessions reviewed and to guarantee that NBS do not (re)create nature-for-elite profit and greenwashing by repackaging past, harmful, nature conservation and restoration, and adaptation programs, we suggest below eight justice-centered principles (Fig. 1) that should govern the present and future of NBS, drawing on Jordan and Lenschow stages of the policy cycle, especially as they refer to environmental policy making (Jordan and Lenschow 2010), and responding to recent calls for addressing implementation challenges and operationalizing NBS principles (Kumar et al. 2020; Wickenberg et al. 2021). Drawing from recent research from colleagues and ourselves, we articulate and dissect these principles in ways that can support decisions for more just NBS-related policy options as well as for the design, implementation, and evaluation phases of a more justice-centered NBS policy or project. We suggest repoliticizing NBS and moving toward critical approaches, beyond calls for inter- and trans-disciplinary approaches meant to rely on holistic co-creation processes and the engagement of a variety of stakeholders across sectors and levels (Kumar et al. 2020).

First, NBS should count with rigorous, ex ante, assessments of their benefits. Rather than assuming benefits a priori, projects should develop a clear assessment of the mitigation and adaptation benefits to be achieved, and of the climate risks and impacts that can be avoided or addressed. For example, tree planting or “green roofing” in cities as a cooling strategy is effective (more at daytime than at nighttime), but the evidence for larger scale cooling effects remains inconclusive (Bowler et al. 2010; Cuthbert et al. 2022). Similarly, active reforestation to rehabilitate degraded rural landscapes can increase the provision of specific ecosystem services, including climate mitigation and soil erosion control, but may not necessarily be a cost-effective strategy compared to natural revegetation (Meli et al. 2017; Honey-Rosés et al. 2018). The use of NBS should also maximize both mitigation and adaptation co-benefits, as several cities and regions have already committed to (Meli et al. 2017; Honey‐Rosés et al. 2018). In Quito, for example, our research shows that NBS projects^4^ under the Climate Action Plan were selected for their positive role in addressing risks of erosion and landslides while protecting and reforesting indigenous lands, thus meeting ecological and social goals.

Second, NBS also need to guarantee regenerative and sustainable economic pathways and confront unsustainable land use patterns. These range from large-scale farming, biofuel production, and mining and other resource extraction in rural areas to large-scale real estate development via densification and urban sprawl, financial developments, visitor- and tourism-driven economies in urban regions. Too many NBS are shown to be deployed without a deep questioning or revisiting of those economic drivers and pathways and coincide side-by-side with growth-generating and land-use change policy options (Kotsila et al. 2021). In contrast, NBS that privilege circular and regenerative economies sustained with care, solidarity, and equity-driven principles can provide sustainable and climate-conscious alternatives. In Portland, Oregon, our research shows how, under a strong climate justice lens, minority-owned cooperatives such as Verde are financing green infrastructure projects to upgrade the homes of Latinx residents and protect them against climate impacts, including heat and flooding (Triguero-Mas et al. 2021). Through Las Adelitas project, Portland cooperatives are also building affordable housing with green features and turning a former abandoned building into secure and green housing for Latinx residents. Both initiatives nurture community wealth creation for racialized groups while also building individual economic power.

Third, NBS must also circumvent the appropriation and/of enclosure of land for greening and conservation. In many countries, nature is enclosed to protect land against either rural deforestation or urban growth, yet in doing so NBS projects also exclude residents from many needed resources, or, at the very least, restrict what uses can be made of certain essential resources for their lives and livelihoods, through development promises (Duffy et al. 2019; Dowie 2011). In addition, while, in many cases, environmental protection laws are being enforced to keep the poor out of protected areas -often violently- (Duffy et al. 2019; Massé 2020), (private) wealth-generating activities or groups are entitled to access natural resources, thus revealing the unequal enforcement of land use regulations (Massé and Lunstrum 2016). Our research also identified such unquestioned dissonances in Medellin, Colombia, whereby the construction of a green belt in the hillslides and slopes of the city strongly regulated the urban growth of informal settlements while leaving high-end real estate developments in El Poblado area untouched and failing to address the needs of the rural–urban border (Isabelle Anguelovski et al. 2019a, b). In sum, NBS should avoid “grabbing” rural and urban landscapes under the discourse of creating new green and resilient cities or landscapes.

Relatedly, NBS should foremost avoid land speculation in both rural and urban areas (and associated land grabbing in agricultural landscapes and green gentrification in cities). With research on NBS increasingly showing how nature is being grabbed by firms, investors, and developers to increase land value and profits, to market new real estate developments, and to close on “green [land] gaps” (García-Lamarca et al. 2022), we argue that NBS projects must be decoupled from speculative and profit-driven dynamics, and rather play a much stronger social role for residents and users, in ways that can secure their needs and responsibilities (Kotsila et al. 2020). For example, in Barcelona, our most recent research identifies that the new 2021–2030 *Pla Natura* and one specific program called *Mans al Verd*^5^ envision the increase of urban green space through the cession of empty lots to residents so that these can be managed and farmed as community gardens. Here, the municipality so far manages to privilege and value residents’ quality of life and biodiversity protection over the sale of unused land to real estate developers. In rural lands, NBS proponents should also learn from the design and implementation principles that explain successful community-driven restoration, sustainable resource management, and conservation initiatives, which range from specific successful examples of UN-endorsed “territories and areas conserved by indigenous peoples and local communities,” known as ICCAs (Eghenter 2018; Ban et al. 2020), to specific policy-driven or project-based initiatives worldwide (Brooks et al. 2012; Dawson et al. 2021).

Fifth, to prioritize environmental values and social objectives, NBS must avoid greenwashing, that is a superficial integration of green objectives, and the privatization of nature for profit. Our recent work demonstrates that too many NBS projects still privilege glitzy greening and building the green image of projects, corporations, cities, or regions while deploying greening projects whose net decarbonization or adaptation gains are rather light. We have previously identified this dynamic as urban green boosterism, that is the construction of a green identity via emblematic and flagship projects, visuals, discourses, and awards that boost the international reputation of cities such as Vancouver, Nantes, Copenhagen, Dublin, or Amsterdam, or Valencia in order to attract new investments (García Lamarca et al. 2021). Yet, in many of these cities, green is rather a brand that is superficially implemented and where nature often becomes privatized. In Dublin, for example, we identified that several green spaces constructed by real estate developers in the working-class neighborhood of The Liberties as public amenities became gated soon after their inauguration in 2019 and were granted permission to be gated by the Dublin City Council despite the original permitting conditions imposed on the site by law (Anguelovski et al. 2021). In some ways, guided by the 2015 Liberties Greening Strategy, the Dublin City Council is working to increase access to green space in the neighborhood by adding new parks such as Bridgefoot Street Park (2022) and Weaver Park (2017). Yet, in practice, we found that those parks are accelerating student- and tourism-led gentrification—with numerous building permits given to student housing and hotel developers denounced by residents—and risk being appropriated by temporary visitors, while smaller, nearby informal green spaces are getting demolished (Anguelovski et al. 2021). In rural lands, a growing body of evidence has also demonstrated that NGOs and other commercial actors are increasingly profiting from conservation (Clements et al. 2016) through the enactment of private protected areas, which are legitimized on the grounds of the current extinction we are facing, and from eco-tourism practices which might result in the marginalization of local social groups and the under valuation of local livelihoods, as shown in recent research in Colombia’s Tayrona National Natural Park (Bocarejo and Ojeda 2016).

Sixth, NBS should be inclusive and empowering, i.e., they should visibilize and recognize the often overlooked, non-expert knowledge of residents and users, especially so for historically marginalized groups, and genuinely include them in the design and management of NBS projects. Otherwise, the needs, vulnerabilities, and identities of such groups risk becoming marginalized while the preferences of higher income or more politically empowered groups get catered to (Anguelovski et al. 2020). In racialized urban contexts in particular, the design, norms, and rules around new green spaces have been shown to overlook the needs of immigrant and minority residents as well as their perceptions of nature and even to increase their control, policing, and exclusion, thus making nature in cities increasingly white (Finney 2014; Kabisch and Haase 2014; Anguelovski and Connolly 2021). In several countries, both in urban and rural regions, conservation and sustainable resource management programs and projects aimed at climate adaptation or mitigation have also been disadvantageous to the poorest and politically disempowered social groups (Nagoda and Nightingale 2017; Ramirez-Reyes et al. 2018; Sovacool 2018; Hoang et al. 2019). In Mexico, for example, our recent research shows mixed results: although the country’s programs of payments for ecosystem services have contributed to halt deforestation and reduce land-use emissions, their design has mostly favored the participation of land-entitled families, which in turn has resulted in unequal distributions of the programs’ incentives at community level (Costedoat et al. 2015; Corbera et al. 2020; Izquierdo-Tort et al. 2022; Jones et al. 2020). In contrast with such exclusionary dynamics, in the broader Cape Town, for example, one promising project we have identified is the Cape Town Environmental Education Trust which tries to address the Apartheid’s legacy of exclusive access to nature for white elites by improving the inclusiveness of urban and peri-urban nature reserves and the effectiveness of biodiversity conservation. Among others, it builds participation pathways for racialized communities traditionally excluded from reserves by fomenting spiritual connections to the spaces, connecting through visitors’ needs and values, and integrating green skills development (Tozer et al. 2020).

As mid- and long- term goal, NBS projects must help tackle long-term green inequalities if they are to fulfill their potential of addressing social and economic objectives in addition to environmental and climate goals. Green inequalities relate here to the lack of opportunity and capacity held by low-income residents to benefit from NBS projects through economic schemes than can support their livelihoods at the individual and community level. In several projects we have examined, NBS are “intentionally” coupled with equity measures. In Washington DC, the 11th Street Bridge Park project—the transformation of a bridge in a greenway with new biodiversity features and recreational opportunities as well as adjacent river clean-up and restoration—is coupled with an Equitable Development plan that funds new minority-owned businesses and social venues, supports affordable housing measures to help avoid displacement, and creates resident-driven greening (Anguelovski et al. 2022).

Last, NBS must also guarantee that people’s relationship with land and nature is repaired and supported. In cities across the US and in rural lands across the Global South, the land of poor and racialized residents has been appropriated through urban segregation and urban renewal policies as well as by large land grabbing practices for conservation, farming, or resource extraction in the countryside (Brockington and Igoe 2006; Sändig 2021). As NBS projects are established to protect nature and land assets for climate and environmental goals, they must also give new rights and reparations to marginalized residents so that land can play an emancipatory function by guaranteeing reproductive and/or productive functions and helping secure economic needs and cultural practices for vulnerable groups. NBS can also play a reparative role, especially so in post-war or conflict contexts, where new public green spaces can help address a violent history and associated socio-spatial trauma and separation. In Berlin, for example, researchers have found that the creation of new large parks has helped the city’s reunification post 1990 by both embracing the city’s historic heritage and making new parks accessible to all residents (Draus et al. 2019). In Colombia, since 2017, we identified that the national Law of payments for ecosystem services (PES) was passed to reinforce the country’s 2016 Peace Accords by promoting publicly funded PES that could support forest conservation and rural development strategies in regions that had suffered historical violence and where illegal crops were grown, and by allowing former guerrilla and paramilitary members (and their families) to become formal recipients of payments (Moros et al. 2020). A few years after their deployment, however, the ad hoc and insufficiently funded implementation of such programs seem to limit their transformative potential (Montes Cortés 2018).

## A new (just) tale for Nature-Based Solutions

We acknowledge that many NBS have been enacted with the best ecological and socio-economic intentions in mind and offer promising results for climate adaptation and mitigation. However, as we have argued above, their prospective benefits should not be taken for granted, as evidence from the past and the present suggest that risks are many and negative impacts can abound. Therefore, the eight principles and associated case examples developed in this paper outline promising approaches and practices for the governance of NBS as a justice-inspired and -centered policy tool in the rural and urban areas where they are enacted. Specifically, we see the principles as safeguards to improve NBS governance frameworks in ways that generate more just processes and outcomes, and avoid that NBS for climate mitigation and adaptation lead to nature-enabled dispossession.

Our cautionary and critical approach to NBS and our plea to make these policies and projects socially just and environmentally effective are being increasingly recognized in socio-ecological research and have also been put forward by others. In October 2021, for example, the House of Lords Science and Technology Committee wrote Alok Sharma, the COP26 President, warning that NBS should not be a substitute for the need to de-carbonize economies and that they should be implemented with the real  partnership of local communities and affected people.^6^ The Committee echoes the fear of environmental NGOS and alliances, such as the World Rainforest Movement,^7^ the Indigenous Environmental Network, or Friends of the Earth, who have called for a “No to Nature Based Solutions,” denouncing both the risks of monoculture tree plantations and industrial agriculture.^8^

In a context in which nature is being produced, enclosed, and governed in increasingly privatized manners and with unequal social impacts, NBS should also challenge the political economy of rural and urban development while guaranteeing that residents enjoy nature’s benefits, enhance their well-being, and access the emancipatory functions that nature and land can and should play for all.

## Footnotes

1 https://www.leaderspledgefornature.org.

2 https://ec.europa.eu/info/research-and-innovation/research-area/environment/nature-based-solutions/research-policy_en#what-is-the-eus-policy.

3 https://www.cliffordchance.com/content/dam/cliffordchance/briefings/2021/07/cop26-nature-based-solutions-to-climate-change.pdf.

4 See a few of those projects here: https://metrodequito.gob.ec/2019/02/25/segunda-arborizacion-en-el-parque-bicentenario/?lang=en.

5 https://ajuntament.barcelona.cat/ecologiaurbana/ca/pla-buits.

6 https://committees.parliament.uk/committee/193/science-and-technology-committee-lords/news/158042/committee-urges-cop26-to-include-naturebased-solutions-in-its-decision-text/.

7 https://wrm.org.uy/actions-and-campaigns/more-than-200-groups-say-no-to-nature-based-solutions/.

8 https://grain.org/en/article/6734-no-to-nature-based-dispossessions.
